# TEleRehabilitation Nepal (TERN) for People With Spinal Cord Injury and Acquired Brain Injury: A Feasibility Study

**DOI:** 10.1177/11795727221126070

**Published:** 2022-10-18

**Authors:** Raju Dhakal, Mandira Baniya, Rosie M Solomon, Chanda Rana, Prajwal Ghimire, Ram Hariharan, Sophie G Makower, Wei Meng, Stephen Halpin, Sheng Quan Xie, Rory J O’Connor, Matthew J Allsop, Manoj Sivan

**Affiliations:** 1 Spinal Injury Rehabilitation Center, Kavre, Nepal; 2 School of Medicine, University of Leeds, Leeds, UK; 3 Sheffield Teaching Hospitals NHS Trust, Sheffield, UK; 4 Leeds Community Healthcare NHS Trust, Leeds, UK; 5 School of Electronic and Electrical Engineering, University of Leeds, Leeds, UK; 6 National Demonstration Centre in Rehabilitation Medicine, Leeds Teaching Hospitals NHS Trust, Leeds, UK; 7 Academic Department of Rehabilitation Medicine, Leeds Institute of Rheumatic and Musculoskeletal Medicine, School of Medicine, University of Leeds, Leeds, UK; 8 Leeds Institute of Health Science, University of Leeds, Leeds, UK

**Keywords:** Telemedicine, rehabilitation, LMIC, chronic conditions, disability, health services, long-term conditions

## Abstract

**BACKGROUND:**

Spinal Cord Injury (SCI) or Acquired Brain Injury (ABI) leads to disability, unemployment, loss of income, decreased quality of life and increased mortality. The impact is worse in Low-and Middle-Income Countries (LMICs) due to a lack of efficient long-term rehabilitative care. This study aims to explore the feasibility and acceptability of a telerehabilitation programme in Nepal.

**METHODS:**

Prospective cohort feasibility study in a community setting following discharge from a specialist rehabilitation centre in Nepal. Patients with SCI or ABI who had previously accessed specialist rehabilitation were connected to a specialist Multidisciplinary Team (MDT) in the centre through a video conference system for comprehensive remote assessments and virtual individualised interventions. Data were captured on recruitment, non-participation rates, retention, acceptability (via end-of-study in-depth interviews with a subset of participants) and outcome measures including the Modified Barthel Index (MBI), Depression Anxiety Stress Scale (DASS) and EuroQol-5D (EQ-5D), completed pre- and post-programme.

**RESULTS:**

97 participants with SCI (n = 82) or ABI (n = 15) discharged from the centre during an 18-month period were approached and enrolled on the study. The telerehabilitation programme facilitated the delivery of support around multiple aspects of rehabilitation care, such as spasticity treatments and pain management. Outcome measures indicated a significant improvement in functional independence (*P* < .001), depression, anxiety and stress (*P* < .001) and quality of life (*P* < .001). Qualitative interviews (n = 18) revealed participants found the programme acceptable, valuing regular contact and input from MDT professionals and avoiding expensive and lengthy travel.

**CONCLUSION:**

This is the first study in Nepal to identify telerehabilitation as a feasible and acceptable approach to augment the provision of specialist rehabilitation. Future research is needed to assess the suitability of the programme for other conditions requiring specialist rehabilitation and determine the mechanisms underpinning improved outcomes for people with SCI or ABI.

**TRIAL REGISTRATION:**

ClinicalTrials.gov Identifier: NCT04914650

## Introduction

The disabilities an individual experiences after spinal cord injury (SCI) or acquired brain injury (ABI) often results in entering a spiral of loss of function, unemployment, loss of income and ill-health.^1-4^ Outcomes are particularly poor in low- and middle-income countries (LMICs) due to the paucity of specialist rehabilitative services.^2^ Despite the inclusion of rehabilitation in the national health policy and planning in Nepal, there is no government hospital providing specialist rehabilitation services in a country with over 26 million people, out of which nearly 2% (513 132 people) are living with disabilities.^5^ There are also currently no established pathways for community-oriented rehabilitation for individuals with a disability after discharge from acute hospital care.^6^ Travelling to hospital services can include multiple challenges for both rural and urban dwellers with disabilities due to the mountainous terrains of the region and limited transport facilities.

Individuals with SCI are known to experience multiple secondary complications, poor community reintegration and high mortality.^7^ A hospital discharge follow-up study in Nepal determined that one-quarter of individuals with SCI died within 1 to 2 years post-discharge, rising to one-third of those who used wheelchairs.^8^ The vast majority had ‘severe’ or ‘extreme’ restrictions to community participation and one-third of survivors were readmitted due to medical complications.^8^ This contrasts with outcomes in several developed countries which have better survival and lower complication rates, in part due to increased provision of community rehabilitation services.^9^

Telemedicine is typically defined as the provision of health care services at a distance, which can include the use of information communication technology (ICT) for medical diagnostics, monitoring and therapeutic purposes.^10^ Broadly, there is emerging evidence for the effectiveness of telemedicine in improving outcomes for patients and is viewed as an acceptable approach by both patients and health professionals.^11^ Mobile telecommunication connectivity in Nepal has grown exponentially in the last decade. In 2020, the proportion of the population accessing broadband in Nepal reached 80%, largely 3G and 4G mobile data services, whilst standard mobile telephone access had coverage for 98% of the population.^12^ Telemedicine, applied to diabetes management and tele-physiotherapy for musculoskeletal problems, has been shown to be effective and cost-saving in rural Nepal.^13,14^

The application of mobile technology in supporting people living with long-term physical and cognitive disabilities and remote delivery of rehabilitative care (also referred to as telerehabilitation) in Nepal, is limited.^15,16^ Our recent systematic review noted that there is limited literature available reporting the use and effectiveness of telerehabilitation as an intervention for people with SCI across all LMICs with a need to establish the changes in patient outcomes arising from the use of telerehabilitation.^17^ There is a need to explore its feasibility, efficacy and cost-effectiveness in Nepal, particularly given the shortage of specialist rehabilitation teams, low rehabilitation resources in the community, limited transportation infrastructure and mountainous terrain of the country. Whilst approaches that utilise digital technology to deliver care show promise in LMICs, an understanding of how these translate to address health system challenges and long-term rehabilitative care is yet to be explored. This project, called TEleRehabilitation Nepal (TERN), aimed to determine the feasibility and acceptability of a telerehabilitation approach for a cohort of individuals with SCI or ABI discharged from a specialist rehabilitation centre, alongside exploring how a telerehabilitation approach influences patient outcomes relating to improvements in functional independence, depression, anxiety and quality of life.

## Materials and Methods

Ethical approval for the study was obtained from the Nepal Health Research Council (reference: 1727) and the University of Leeds School of Medicine Research Ethics Committee (reference; MREC 19-031) and the feasibility study protocol registered with clinicaltrials.gov (ClinicalTrials.gov Identifier: NCT04914650). Project activities were conducted in accordance with the principles outlined in the Helsinki Declaration. All participants provided written informed consent before participation in the study. Reporting is aligned with the STROBE checklist for observational studies.

### Setting and participants

The Spinal Injury Rehabilitation Centre (SIRC) in Nepal, located near the capital city of Kathmandu, is a non-governmental organization (NGO) providing inpatient and outpatient care for individuals with SCI or ABI. The services offered at SIRC include medical care, nursing, physiotherapy, occupational therapy, psychology, peer counselling, vocational training, social services, community-based rehabilitation, prosthesis, orthosis and assistive devices and other extended services (such as speech therapy and recreational therapies). The TERN study was set up through a collaboration between SIRC and the Academic Department of Rehabilitation Medicine at the University of Leeds in the United Kingdom.

This was a prospective cohort feasibility study to explore the acceptability and feasibility of the telerehabilitation programme and its impact on the outcomes of people with SCI and ABI to facilitate follow-up care after discharge. The inclusion criteria included patients at SIRC who were: (i) age 18 years or above; (ii) individuals with a diagnosis of SCI or ABI (diagnosed by clinical assessment and imaging in the acute hospital before transfer to SIRC) who received inpatient specialist rehabilitative care in SIRC (SCI included both traumatic and non-traumatic cases and ABI included traumatic brain injury, brain tumour and stroke); (iii) those discharged from SIRC between February 2018 and August 2019, irrespective of any length of stay at SIRC; (iv) those living within 1 day of travel distance from SIRC. Exclusion criteria included individuals who did not report any ongoing rehabilitation needs when approached by the research team or those not willing to participate in the study.

### Sampling and data collection procedure

The sample size was determined for the purpose of informing a future study and intervention development. We sought to recruit a sample size of at least 50 participants to allow for meaningful and reliable data which could be used to power future trials.^18^ Sample sizes between 24 and 50 have been recommended for feasibility studies^19,20^ although we sought to increase this number where possible given the novel application of the technology for rehabilitation care in the context of Nepal. We gathered data on recruitment, non-participation rates, retention and acceptability (via in-depth interviews) and reported on findings from outcome assessments captured as part of the feasibility study. To identify a study cohort, from a list of 250 consecutively discharged participants between February 2018 and August 2019, a list was prepared of 129 probable participants living at a distance of a maximum of 1-day of travel from SIRC. An electronic list was created in Microsoft Excel from paper-based records and stored securely on a laptop with access enabled only to the research team. Eligible participants were contacted by telephone to discuss the study and obtain verbal consent if they were suitable and willing to participate. The participant information sheet and consent form were read to potential participants over the phone. After gaining verbal consent, a home visit by a member (social worker) of the SIRC team was planned for each participant within 1 month. On the home visit, the social worker explained the nature of the study and obtained written consent. The social worker completed a baseline assessment with participants, ensuring participants understood the content and items to support accuracy in responses. This included gathering participant demographic characteristics including age, sex, distance from SIRC, terrain (ie, physical features of the geographical area in which the participant resided), nature of the disability, duration since injury, history of consultation at other hospitals since discharge, employment, marital status before and after disability and ability to leave the house. Participants also completed 3 measures at baseline, as described in Table 1 (ie, the Modified Barthel Index [MBI], Depression Anxiety Stress Scale [DASS] and EuroQoL 5 [EQ-5D-5L]). Following the baseline assessment measures, the social worker supported the participant to connect on their mobile phone device to SIRC via the telerehabilitation system. The mobile phone devices held by patients varied as their own personal phones were used. Common models included those manufactured by Xiaomi, Samsung and Huawei which were being widely used at the time of the study. The outcome measures were available in both English and Nepali languages and participants could choose the language they were comfortable with. For those with cognitive difficulties, primary caregivers or family members supported participants by reading aloud the content of the information sheet. Primary caregivers or family members also read aloud information relating to informed consent. Where both patients and caregivers were unable to read and write, the information sheet was read aloud to them both and explained by a member of the social worker. Prior to data collection commencing by the social worker, training was provided by the wider research team to enable orientation with the data collection tools and protocols for their use and completion with study participants.

**Table 1.** Overview of outcome measures.

Outcome measure	Description of measure
Modified Barthel Index (MBI)	MBI measures the performance of an individual in activities of daily living (ADL) that is, feeding, grooming, bathing, dressing, continence of bowel and bladder, transfer to and from a wheelchair, transferring to and from a toilet, use of a wheelchair, use of stairs and walking. The items are scored based on the amount of physical assistance required to perform the task. Each item has 5 categories, the first category indicates full dependence, and the fifth category indicates full independence. The total score ranges from 0 to 100 where a higher score indicates increased independence in performing ADLs.^21^ The tool has been shown to be valid and reliable in individuals with SCI.^22^
Depression Anxiety Stress Scale (DASS)	DASS-21 is a self-report measure derived from a 42-item DASS scale. DASS-21 consists of three 7-item subscales: depression, anxiety and stress. The items refer to the feelings that occurred during the past week. Each item is scored on a 4-point scale (0 = ‘did not apply to me at all’, to 3 = ‘applied to me very much or most of the time’). The score for each subscale ranges from 0 to 21, where a higher score indicates greater severity. DASS-21 is a valid, reliable and easy to use tool; the total scores range between 0 and 63.^23^
EuroQoL 5 (EQ-5D-5L)	EQ-5D-5L was used as a measure of assessing the health-related quality of life (HRQoL) of the participants. It is a 5-level version of EuroQoL. It comprises of 5 dimensions of health: mobility, self-care, usual activities, pain/discomfort and anxiety/depression; each dimension is scored on a 5-point scale (1 = ‘no problem’-5 = ‘unable to/extreme problem’). In addition, the visual analogue score (EQ-VAS) was used to measure the direct valuation of the current state of health of participants on a 0 to 100 scale, where ‘0’ refers to ‘the worst health you can imagine’ and 100 refers to ‘the best health you can imagine’. This tool has shown excellent psychometric properties amongst the SCI and ABI population.^24^ The EQ-5D index value was derived from the 5 dimensions of EQ-5D-5L and was calculated using the EQ-5D-5L Crosswalk Index Value Calculator.^25^ The EQ-5D index value ranges from 0 to 1, where 0 indicates severely ill and 1 indicates perfect health.

The telerehabilitation system comprised of a specialised audio-visual system that was installed in the multi-disciplinary team (MDT) room at SIRC to facilitate remote consultations. The system included a smart 43-inch smart LED television, People link UVC (conference call speaker), People link Quordo (conference phone), People i-Com WHD camera (a web camera). All devices were connected through a laptop. The telerehabilitation consultation was provided using an online video conferencing platform, InstaVC. In cases where InstaVC could not be used, social media platforms or audio conferencing, via mobile phones, were used. The social media platforms used included Facebook Messenger, WhatsApp and Viber.

The telerehabilitation team at SIRC is comprised of an MDT of 6 members including a rehabilitation physician, rehabilitation nurse, physiotherapist, occupational therapist and social worker. During a participant’s first session they met with the MDT via the telerehabilitation system. The MDT discussed the ongoing physical, cognitive, psychological and vocational problems the individual was experiencing. The problems that could be intervened at home using local resources were immediately addressed during the consultation. This included advice on medications, skincare, catheter care, exercises, use of assistive and mobility aids, dietary plans and counselling on general coping skills. Equipment including walking aids, catheters, mattresses or hospital beds were arranged to be delivered in some cases. The team had the option of referring participants to the nearest hospital for specific diagnostics and treatment or admitting them to SIRC if needed. Some participants needed more than one follow-up consultation to complete the interventions, review the goals and complete the outcome measures. During the first session, desired goals were discussed and decided through discussion between the MDT and the participant. The goals could be related to reviewed physical, cognitive, psychosocial or vocational aspects for each participant depending on areas they preferred to have input from the MDT.

Following completion of baseline measures, for each individual, video or telephone consultations were completed 1 to 2 times over a week. Access to the MDT via the telerehabilitation system continued until a participant achieved the desired goals. A follow-up assessment of a participant was completed within 4 weeks of completion of the telerehabilitation programme by a social worker. During the follow-up assessment, measures taken at baseline (ie, the MBI, DASS and EQ-5D-5L) were repeated alongside an additional 5-point Likert scale that was used to assess the perception of participants about the benefit(s) of telerehabilitation. The scale ranged from ‘1’ completely disagree to ‘5’ completely agree that telerehabilitation was beneficial. At post-intervention, a subset of participants was purposively selected by participants’ sex (male and female), residential location (rural or urban) and type of disability (SCI vs ABI). In-depth qualitative interviews were conducted by members of the research team (MB, CR) to explore participant experiences and acceptability of the telerehabilitation intervention. Similar to outcome assessment, primary caregivers or family members were involved in the interviews for participants with cognitive difficulties to facilitate communication with the research team. Interviews were audio recorded and transcribed verbatim.

### Data analysis

Frequency counts were used to summarise the demographic and injury-related characteristics of the participants. Binary and categorical data are presented as frequencies and percentages and continuous variables as a mean and standard deviation (SD). The normality of the dependent variables was determined by assessing skewness and kurtosis. A paired sample *t*-test or Wilcoxon signed-rank test was applied to compare the pre and post-intervention data. The significance level was considered at a *P*-value <.05. Cohen’s *d* effect sizes were used. Data were analysed using SPSS (SPSS: Version 20.0. Chicago, IL, USA). Transcripts from in-depth interviews were independently coded by members of the research team (MB, CR). Reflexive thematic analysis^26,27^ was applied to inductively generate themes from the qualitative data. NVivo software was used to organise the qualitative data.

## Results

**Figure 1.**
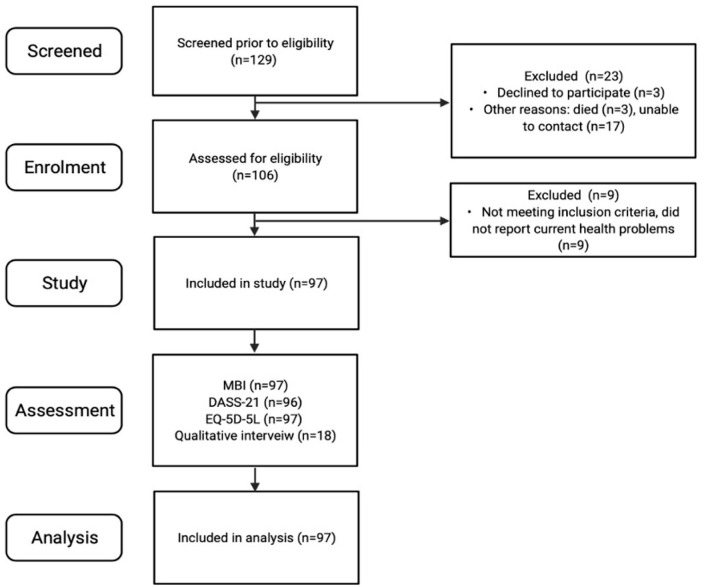
Flowchart of number of participants approached and included.

Among 129 potentially eligible participants identified, 97 were recruited and participated in the study. Figure 1 outlines reasons for non-participation, including participants who could not be contacted by phone (n = 17), reported no current health concerns (n = 9), had died (n = 3) or declined to participate (n = 3). All 97 participants completed the telerehabilitation programme including post-programme assessments and were retained to the end of the study period. Less than half of the participants (43.3%) were able to leave their house without assistance at the first assessment (Table 2). In total, 14 participants (all with ABI) required support from a family member of caregiver to complete study questionnaires and participate in a semi-structured interview due to limited communication. An overview of the TERN telerehabilitation intervention and adaptations made during the study is outlined in Table 3 aligned with the template for intervention description and replication (TIDieR)^28^ checklist.

**Table 2.** Demographic and disability-related characteristics of the participants (n = 97).

Variables	N (%)
Age (years) mean = 38.4, SD = 13.2, Min-Max = 18-73	
Sex	
Female	20 (20.6)
Male	77 (79.4)
Province	
Province No. 1	21 (21.6)
Madhesh	11 (11.3)
Bagmati	42 (43.3)
Gandaki	4 (4.1)
Lumbini	12 (12.4)
Karnali	1 (1.0)
Sudurpashchim	6 (6.2)
Terrain	
Plain lands	41 (42.3)
Hilly	56 (57.7)
Nature of disability	
ABI	15 (15.5)
SCI	82 (84.5)
Type of SCI (n = 82)	
Tetraplegia	18 (22.0)
Paraplegia	64 (78.0)
Marital status before injury	
Single	15 (15.4)
Married	80 (82.5)
Divorced	1 (1.0)
Widower	1 (1.0)
Marital status after injury	
Single	15 (15.5)
Married	76 (78.4)
Separated	2 (2.1)
Divorced	3 (3.1)
Widower	1 (1.0)
Employment status before injury	
Unemployed	10 (10.3)
Employed	84 (86.6)
Retired	3 (3.1)
Employment status after injury	
Unemployed	80 (82.5)
Employed	13 (13.4)
Retired	4 (4.1)
Treated in other hospitals after discharge	
Yes	37 (38.1)
No	60 (61.9)
Ability to leave the house after injury	
No, confined to home	17 (17.5)
With assistance	38 (39.2)
Without assistance	42 (43.3)

**Table 3.** Intervention description aligned with the Template for Intervention Description and Replication (TIDieR) checklist.

Brief name	TEleRehabilitation Nepal (TERN)
WHY	The intervention sought to connect to previously discharged patients remotely to enable the provision of suggestions/solutions for their current health problems. Broadly the intervention sought to increase independence in activities of daily living and improve the quality of life of participants.
WHAT	Materials: Exercise and transfer technique videos, assistive devices (wheelchair, ankle or knee orthosis, walker) cushion, medicines, intermittent/Foley’s catheters
	Procedures: Video conferences for demonstration of exercises, transfer techniques, advice on bladder and bowel care, pressure sore care, medication and assistive devices prescription and counselling. In case of no or poor internet, advice was given through voice call on a mobile phone consultation. Centre visits as outpatients were used to collect assistive devices, wheelchairs and other necessary items (eg, catheters, medicines).
WHO PROVIDED	Rehabilitation physician, physiotherapist, rehabilitation nurse, occupational therapist
HOW	Interventions were made possible through teleconsultation that was provided based on the needs of participants. Telecommunication platforms were used to facilitate video calls. During the study these included: InstaVC (n = 31), Facebook messenger (n = 40), WhatsApp or Viber (n = 21). Telephone audio call only (n = 21).
WHERE	Interventions delivered by a healthcare team based at SIRC, with participants typically at their home or in the community setting
WHEN and HOW MUCH	For each individual, video or telephone consultations were completed 1 to 2 times over a week. A follow-up assessment of the individual was done at 1 to 4 week following completion of the telerehabilitation programme.
TAILORING	The telerehabilitation team at SIRC is comprised of a multidisciplinary team of 6 members including a rehabilitation physician, rehabilitation nurse, physiotherapist, occupational therapist and social worker. During an initial telerehabilitation session, the team discussed the ongoing physical, cognitive, psychological and vocational problems the individual was experiencing. The problems that could be intervened at home using local resources were immediately addressed during the consultation. This included advice on medications, skincare, catheter care, exercises, use of assistive and mobility aids, dietary plans and counselling on general coping skills. Equipment including walking aids, catheters, mattresses or hospital beds were arranged to be delivered in some cases. The team had the option of referring participants to the nearest hospital for specific diagnostics and treatment, undertaking a home visit to deliver interventions, or admitting them to SIRC if needed. Some participants needed more than one follow-up consultation to complete the intervention, review the goals and complete the outcome measures.
HOW WELL	Among 129 potentially eligible participants identified, 97 were recruited and participated in the study. All participants completed the telerehabilitation programme including post-programme assessments and were retained to the end of the study period. Participants were willing to engage with the telerehabilitation approach but challenges were experienced. These included: (i) Only 34% of study participants owned a smartphone; (ii) Unreliable internet connections were commonly experienced; (iii) Internet data provided a more reliable connection but cost-prohibitive for participants and (iv) Pre-filmed videos for common topics were preferred over live demonstrations of exercises. Adherence was affected by (i) caregivers having difficulty in assisting to complete the intervention (eg, the only family member was unwell and could not perform catheterisation); (ii) they were unwilling to carry out requested intervention and (iii) prescribed medications were either not available in the local pharmacy or the participant did not want to take medicines because reportedly it was not effective to reduce pain and/or spasticity.

**Figure 2.**
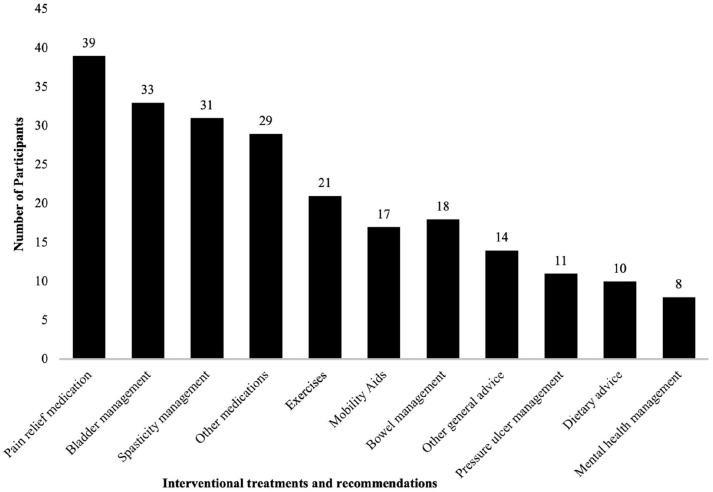
The number of participants who received each of the interventional recommendations.

Of all the participants, 13 required more than one telerehabilitation consultation. This included for changes in pain medication (n = 5), additional exercises (n = 4) and a bladder/bowel assessment (n = 4). The range of interventions delivered as part of the telerehabilitation programme for all participants is summarised in Table 4 and Figure 2. Among 68 (70%) participants who were asked (on a Likert scale) about the benefit of telerehabilitation, 51 (51/68; 75.0%) agreed and 17 (17/68; 25.0%) completely agreed that telerehabilitation was beneficial. Face-to-face interviews with a subset of participants (n = 18) outlined reports of the telerehabilitation approach being acceptable alongside participants providing recommendations for how the implementation of telerehabilitation could be refined in future development (key findings from qualitative interviews are listed in Table 5).

**Table 4.** Interventional recommendations provided during telerehabilitation.

Type of intervention	Number of participants	Details
Pain relief	41	Medication: Anticonvulsants—Pregabalin (n = 37), Gabapentin (n = 1); NSAIDs—Aceclofenac (n = 12), Etoricoxib (n = 2); Tricyclic Amitriptyline (n = 11)
		Physical modalities (heat pack) (n = 1)
Exercises	21	Progressive resistance n= and passive range of motion exercises for upper and lower limbs (n = 26), back (n = 3), pelvic floor (n = 4)
		Passive range of motion (n = 5), Tenodesis grip (n = 1)
Spasticity management	31	Baclofen (n = 31), Tizanidine (n = 13)
Other medications	30	Proton pump inhibitors—Omeprazole (n = 28); H2 blocker-Ranitidine (n = 1)
		Antibiotics—Amoxyclav (n = 1), Cefixime (n = 1); Vitamin supplements – Calcium, Vitamin D3 and Vitamin B12 (n = 21); Zolpidem (n = 1); Rotacap Salmeterol and Fluticasone inhaler (n = 1); Anti-emetic-Domepridone (n = 1)
Assistive products/ADL aids	10	Commode chair (n = 9), modified spoon (n = 1)
Mobility aids	17	Ankle Foot Orthosis (n = 3), Wheelchair (n = 2), Knee brace orthosis (n = 1), Walker (n = 1)
Bladder management	33	Medication: Anticholinergics-Tolterodine (n = 18), Oxybutynin (n = 1), antibiotics (n = 1), tricyclic antidepressant (n = 2), urine alkalyzer (n = 2); Investigations - urine routine and microscopy (n = 2), ultrasound kidney, ureter, bladder (n = 2)
		Bladder catheterisation—clean intermittent self-catheterisation n = 10, indwelling n = 1; Bladder diary (n = 5)
Bowel management	19	Medication—Bisacodyl (n = 10), lactulose (n = 6), Cremaffin syrup (n = 5); Herbal ointment (n = 5) for haemorrhoids
		Bowel training advice (n = 5), bed pan (n = 1)
Pressure ulcer	11	Pressure relief positioning (n = 9), wound dressing (n = 11)
Dietary advice	11	High protein diet (n = 6), high fibre diet (n = 3), low calorie diet (n = 2), general dietary advice (n = 2)
Mental health management	8	Medication—tricyclic anti-depressant-amitriptyline (n = 4), beta-blocker-propanolol (n = 1)
		Psychological counselling (n = 1)
Sexual and reproductive advice	2	Counselling (n = 2); Medication—Sildenafil (n = 1)
Other general advice	14	ADLs and self-care advice: transfer (n = 5), dressing (n = 2), bathing (n = 1)
		Gait evaluation and advice (n = 3)
		Referral—Ophthalmology (n = 1), X-ray (n = 2)

**Table 5.** Summary of key thematic aspects of data gathered during in-depth interviews.

Theme	Summary	Supporting quotes
Receptiveness to and acceptability of telerehabilitation	All participants were receptive to the concept of telerehabilitation. Most participants lived a long distance from SIRC with the approach affording avoidance of long, difficult journeys. Buses and taxi drivers are often reluctant to transport wheelchair users, caused by what was perceived as an aversion to potential risks of transporting a person with a disability. Participants reported that travelling by typically inaccessible public transport is burdensome and creates hassle which can be avoided with telerehabilitation. An alternative option is to travel by ambulance, but this mode is very expensive and often cost-prohibitive for participants.	*‘This is an extremely important step taken because I feel being a wheelchair user, with poor accessibility we cannot even access the basic health needs. In this condition to be able to connect back to SIRC team will ease so many hassles of transportation and be able to access teleconsultation from home will ease our life to great extent’.* Male, SCI, urban community
Overcoming challenges to access irrespective of region	Participants from rural areas expressed the benefit of being able to consult staff at SIRC to determine whether a problem being experienced needs immediate management or can be addressed over the coming days. This was seen as a way of decreasing the stress experienced when problems arise.	*‘To my understanding, rather than travelling all the way to SIRC. . . we can talk on phone or contact via phone and share our problems. We can get the right suggestions whether it is a real problem or not to be worried. We can wait for a week or need immediate admission to a health center’.* Male, SCI, rural community
	Participants shared that even in urban areas, it is difficult to access health services. They considered telerehabilitation as a useful means of accessing health services during emergencies from home.	*‘I am very positive about it. Although we live in a city area, we do not know many things about the management of various health problems. It must be more difficult in rural areas. For emergency help it is very helpful. You have started a very good programme. I appreciate this initiative’.* Female, stroke, urban community
Implementation preferences	Participants from urban areas requested planned online meetings to accommodate family members’ jobs. For example, by selecting a fixed topic or topics for discussion for a planned day where patient participants or their caregivers would then be able to present any challenges being experienced in real-time to the rehabilitation team.	*‘I think it would be better to have a specific day, time, and specific topic for discussion as part of the telerehabilitation programme that you are going to start. If there is a team that is available maybe on Saturday or Sunday afternoon for an hour with a pre-informed topic for discussion, we can arrange our time to attend the tele meeting and discuss our problems’.* Caregiver (of participant with stroke), female, urban community
	While telerehabilitation was seen as a way that participants may be able to decrease institutional visits, participants also expressed the value placed on being met face-to-face in the home. This was felt necessary to identify problems that may remain hidden through a mobile phone and enable participants to feel more comfortable to share their problems.	*‘I would also suggest thinking about the possibility to meet the patients in person through home visits, may be once every two to three months. There is a different feeling in the patients and the family members like us when we see the doctor or other professionals in white coat. Patients and caregivers tend to evolve with the problems that they never told to each other. There is an environment or the confidence about your problems to be heard and solved as you see the concerned health professional and you want to talk to them about what you are facing or feeling exactly rather than only doing exercises at home by ourselves or talking via phone’.* Caregiver (of participant with stroke), female, urban community
	Another service requested by some participants residing in urban areas was for support with procuring relevant online information that may be useful for their condition. According to 1 participant, even though they referred to online websites for information about the management of common problems of stroke, they did not appear reliable. If SIRC could help to identify and curate information on the identification and management of common problems at home, they would feel more confident and trust the resources.	*‘If SIRC can develop a mobile application about common problems and therapies related to stroke or spinal injury, we can be updated about various symptoms, how to improve speech, about monitoring the progress in our patients and many more things. We use you tube to learn about such things, but we cannot be assured about the reliability and authenticity of the information from you tube. In that aspect, whatever information comes from SIRC will be reliable for us’.* Male, SCI, urban community

Multiple learning points were derived from discussions with participants that can be used to guide the future development of telerehabilitation in Nepal (see Table 5 for a summary of findings from face-to-face interviews). Challenges experienced by participants included only 34% of study participants owning a smartphone, and unreliable internet connections were commonly experienced. Whilst internet data provided a more reliable connection it was expensive and most participants could not afford to use this method. Problems were also encountered while using video demonstrations for interventions such as exercises and transfer techniques. Participants reported that they preferred pre-filmed videos for common topics, despite the use of a specialised video conferencing system. This was easier for participants to watch and understand and saved time during consultations so that other issues could be addressed. Adherence to prescribed interventions was a limitation for some participants. The reasons included: (i) caregivers having difficulty in assisting to complete the intervention (eg, the only family member was unwell and could not perform catheterisation); (ii) they were unwilling to carry out requested interventions and (iii) prescribed medications were either not available in the local pharmacy or the participant did not want to take medicines because reportedly it was not effective to reduce pain and/or spasticity.

The scores for severity of depression, anxiety and stress for participants with SCI or ABI significantly decreased after intervention (*P* < .01) and the EQ-5D index score significantly increased post-test with *P* < .001 (Wilcoxon signed-rank test) (Tables 6 and 7). There was a significant mean difference (*P* < .001) between the pre-and post-intervention MBI and the visual analogue scale included as an item of the EQ-5D-5L with effect sizes −0.4 and −0.7 respectively (Table 6).

**Table 6.** Pre- and post-intervention DASS and EQ-5D-5L domain scores.

Variables	Pre-intervention n (%)	Post-intervention n (%)	*P-*value^a^
DASS-21 domains			
Depression (n = 96)			<.001
Normal	66 (68.8)	68 (70.1)	
Mild	6 (6.3)	10 (10.3)	
Moderate	16 (16.7)	13 (13.4)	
Severe	3 (3.1)	—	
Extremely severe	5 (5.2)	5 (5.2)	
Anxiety (n = 96)			.001
Normal	87 (90.6)	91 (94.8)	
Mild	5 (5.2)	2 (2.1)	
Moderate	2 (2.1)	1 (1.0)	
Severe	—	—	
Extremely severe	2 (2.1)	2 (2.1)	
Stress (n = 96)			<.001
Normal	77 (80.2)	85 (88.5)	
Mild	10 (10.4)	4 (4.2)	
Moderate	5 (5.2)	4 (4.2)	
Severe	2 (2.1)	1 (1.0)	
Extremely severe	2 (2.1)	2 (2.1)	
EQ-5D-5L domains			
Mobility			.006
No problem	32 (33.0)	36 (37.1)	
Slight problem	34 (35.1)	35 (36.1)	
Moderate problem	15 (15.5)	13 (13.4)	
Severe problem	5 (5.2)	5 (5.2)	
Unable to walk	11 (11.3)	8 (8.2)	
Self-care			<.001
No problem	32 (33.0)	36 (37.1)	
Slight problem	12 (12.4)	12 (12.4)	
Moderate problem	23 (23.7)	25 (25.8)	
Severe problem	19 (19.6)	14 (14.4)	
Unable	11 (11.3)	10 (10.3)	
Usual activities			.004
No problem	44 (45.4)	49 (50.5)	
Slight problem	29 (29.9)	26 (26.8)	
Moderate problem	9 (9.3)	9 (9.3)	
Severe problem	8 (8.2)	7 (7.2)	
Unable	7 (7.2)	6 (6.2)	
Pain/discomfort			<.001
No pain	14 (14.4)	15 (15.5)	
Slight pain	28 (28.9)	47 (48.5)	
Moderate pain	24 (24.7)	31 (32.0)	
Severe pain	24 (24.7)	4 (4.1)	
Extreme pain	7 (7.2)	—	
Anxiety/depression			<.001
Not anxious	27 (27.8)	33 (34.0)	
Slightly anxious	35 (36.1)	39 (40.2)	
Moderately anxious	17 (17.5)	17 (17.5)	
Severely anxious	16 (16.5)	6 (6.2)	
Extremely anxious	2 (2.1)	2 (2.1)	

Abbreviations: DASS-21, Depression, Anxiety, Stress Scale 21; EQ-5D-5L, EuroQol 5 domains.

a Wilcoxon signed rank test.

**Table 7.** Mean difference between pre and post-test MBI scores EQ-VAS score.

Variables	Mean	SD	Mean difference	*P*-value^a^	Cohen’s *d* effect size	95% CI	
						Lower	Lower
MBI total							
Pre-intervention	60.9	27.3	−1.5	<.001	−.4	−0.60	−0.19
Post-intervention	62.4	27.3					
EQ-5D VAS							
Pre-intervention	60.0	25.7	−5.7	<.001	−.7	−0.92	−0.47
Post-intervention	65.6	22.3					

Abbreviations: EQ-5D VAS, EuroQol 5 Domains Visual Analogue Scale; MBI, modified Barthel Index (SHAH version).

a Paired sample *t*-test.

### Unanticipated care issues identified through the telerehabilitation approach

Two participants had to stop taking Pregabalin, which was prescribed to manage their neuropathic pain because it made them too drowsy. One participant experienced increased pain lying in the prone position because of fractured ribs. One participant sat in an unrecommended position in his wheelchair. When the team made recommendations for his sitting position, he found it more uncomfortable but relieved the pain by returning to his original sitting position.

## Discussion

This study assessed the feasibility of a telerehabilitation approach for follow-up and supporting intervention remotely for people living with physical, cognitive and psychological problems from SCI or ABI in Nepal. The findings suggest that a telerehabilitation approach may increase independence in carrying out activities of daily living and improve psychological health and quality of life among people with ABI or SCI. In addition, most participants reported the telerehabilitation approach was beneficial and in qualitative interviews highlighted it is an acceptable approach that can help to address health needs. Interviews also determined the requirements of patients and their caregivers that can be used to guide future iterations and implementation of telerehabilitation approaches in Nepal.

In Nepal, the current post-discharge rehabilitation care for individuals with SCI and ABI is not meeting individuals’ needs.^6^ There is a lack of qualified rehabilitation service providers and the cost and burden of transportation for individuals with physical disabilities is high. Individuals have ongoing health problems, such as bladder/bowel issues, pain, spasticity, pressure injuries and psychological problems.^29^ Currently, the majority of people with SCI and ABI in Nepal are discharged directly to their homes after acute management with minimal advice about a long-term rehabilitation plan. In the context of Nepal, only a small number of ABI and SCI survivors can access rehabilitation services. Barriers to this include resource limitation (i.e., only one specialist rehabilitation provider for the entire country), geographical barriers (eg, access from rural and hilly terrains) and lack of awareness about rehabilitation services.^6^ The findings of this study suggest telerehabilitation may provide a feasible and acceptable means of providing follow-up care, and addressing a proportion of unmet rehabilitation needs within the country. To further support this position, the coronavirus disease 19 (COVID-19) pandemic occurred after the study had been set up and was actively recruiting. The telerehabilitation programme subsequently proved useful and remained in place during the pandemic, enabling people to continue to access support and be managed remotely. The SIRC team were able to augment the remit of those who could access the telerehabilitation approach outside the study, enabling continued care to overcome restrictions in place during the COVID-19 pandemic.^30^

Our findings contribute to an increasing evidence base for highlighting the potential for telerehabilitation to improve physical and psychological outcomes for people living with physical disabilities,^31^ multiple sclerosis^32^ and musculoskeletal pain^14^ in the context of Nepal. However, there is widespread variation in how telerehabilitation can be delivered, including the type and duration of interventions provided. In our study, we delivered need-based interventions, with no fixed duration of coaching for each intervention provided, mostly addressing common issues related to pressure point care, bowel/bladder management, pain, exercises, deconditioning and nutrition. This is aligned with need-based approaches to telerehabilitation that have been explored in the USA but not in the context of LMICs.^33^ Future iterations of the approach may need to explore specific criteria or decision-making tools to standardise the selection of interventions that are suitable for delivery via telerehabilitation. In the context of LMICs, telerehabilitation interventions need to be context-specific, often ensuring they are simple, robust and user-friendly for easy operability by less sophisticated technology and with locally available resources. Findings from this study suggest that telerehabilitation is a potentially acceptable and feasible approach to enhance the delivery of rehabilitation care in Nepal. However, future research is required to determine the underlying mechanisms and components of the telerehabilitation approach that were driving improved outcomes for patients. Future theoretical development of the telerehabilitation approach may also seek to explore its ability to support the management of additional conditions also managed by specialist rehabilitation, such as cognitive impairments. This would enable exploration of the extent to which telerehabilitation could be embedded across the provision of specialist rehabilitation services in the country.

The study has limitations. Participants were not followed up over a long-term period after the end of the study, which would inform us on any legacy impact of the telerehabilitation approach. The outcome measures used were self-reported, which may have introduced response bias, particularly given that participants were unblinded to the nature of the study. It is difficult to present differential effects depending on the nature of injury (ABI vs SCI) due to the small sample size. We anticipate that providing such interventions to those with severe cognitive problems and those who lack capacity will be challenging and needs to be explored in future studies. Despite such limitations, the study was sufficient to inform the feasibility and acceptability of the telerehabilitation approach and can inform future testing in a randomised controlled trial and further development of the interventions.

## Conclusion

This is the first study in Nepal to explore the application of telerehabilitation in the provision of follow-up care to individuals with SCI or ABI. This study provides evidence of the feasibility and acceptability of telerehabilitation for these patient groups. The telerehabilitation approach led to improved patient outcomes, and overcame geographical barriers to healthcare access with no observed side effects or risks reported during the study.. Future research is needed to explore the extent to which other long-term conditions requiring specialist rehabilitation can be supported by telerehabilitation, alongside determining the underlying reasons for improved outcomes in patients with SCI or ABI observed in this study.

## Supplemental Material

sj-docx-1-rpo-10.1177_11795727221126070 – Supplemental material for TEleRehabilitation Nepal (TERN) for People With Spinal Cord Injury and Acquired Brain Injury: A Feasibility Study Click here for additional data file. Supplemental material, sj-docx-1-rpo-10.1177_11795727221126070 for TEleRehabilitation Nepal (TERN) for People With Spinal Cord Injury and Acquired Brain Injury: A Feasibility Study by Raju Dhakal, Mandira Baniya, Rosie M Solomon, Chanda Rana, Prajwal Ghimire, Ram Hariharan, Sophie G Makower, Wei Meng, Stephen Halpin, Sheng Quan Xie, Rory J O’Connor, Matthew J Allsop and Manoj Sivan in Rehabilitation Process and Outcome

sj-docx-2-rpo-10.1177_11795727221126070 – Supplemental material for TEleRehabilitation Nepal (TERN) for People With Spinal Cord Injury and Acquired Brain Injury: A Feasibility Study Click here for additional data file. Supplemental material, sj-docx-2-rpo-10.1177_11795727221126070 for TEleRehabilitation Nepal (TERN) for People With Spinal Cord Injury and Acquired Brain Injury: A Feasibility Study by Raju Dhakal, Mandira Baniya, Rosie M Solomon, Chanda Rana, Prajwal Ghimire, Ram Hariharan, Sophie G Makower, Wei Meng, Stephen Halpin, Sheng Quan Xie, Rory J O’Connor, Matthew J Allsop and Manoj Sivan in Rehabilitation Process and Outcome
